# Supplementary material for: DMRforPairs: identifying Differentially Methylated Regions between unique samples using array based methylation profiles
Source: BMC Bioinformatics. 2014 May 15;15:141. doi: 10.1186/1471-2105-15-141 (PMC4046028; doi:10.1186/1471-2105-15-141)
Supplement: Additional file 2 — DMRforPairs output for the comparison of A431-MCF7 and NA17018-NA17105. Please start from the HTML files in each folder. Available via the BMC Bioinformatics website. [file 1471-2105-15-141-S2.zip › 1394847754114233_MOESM2_ESM/A431_MCF7/figures/6727.pdf]

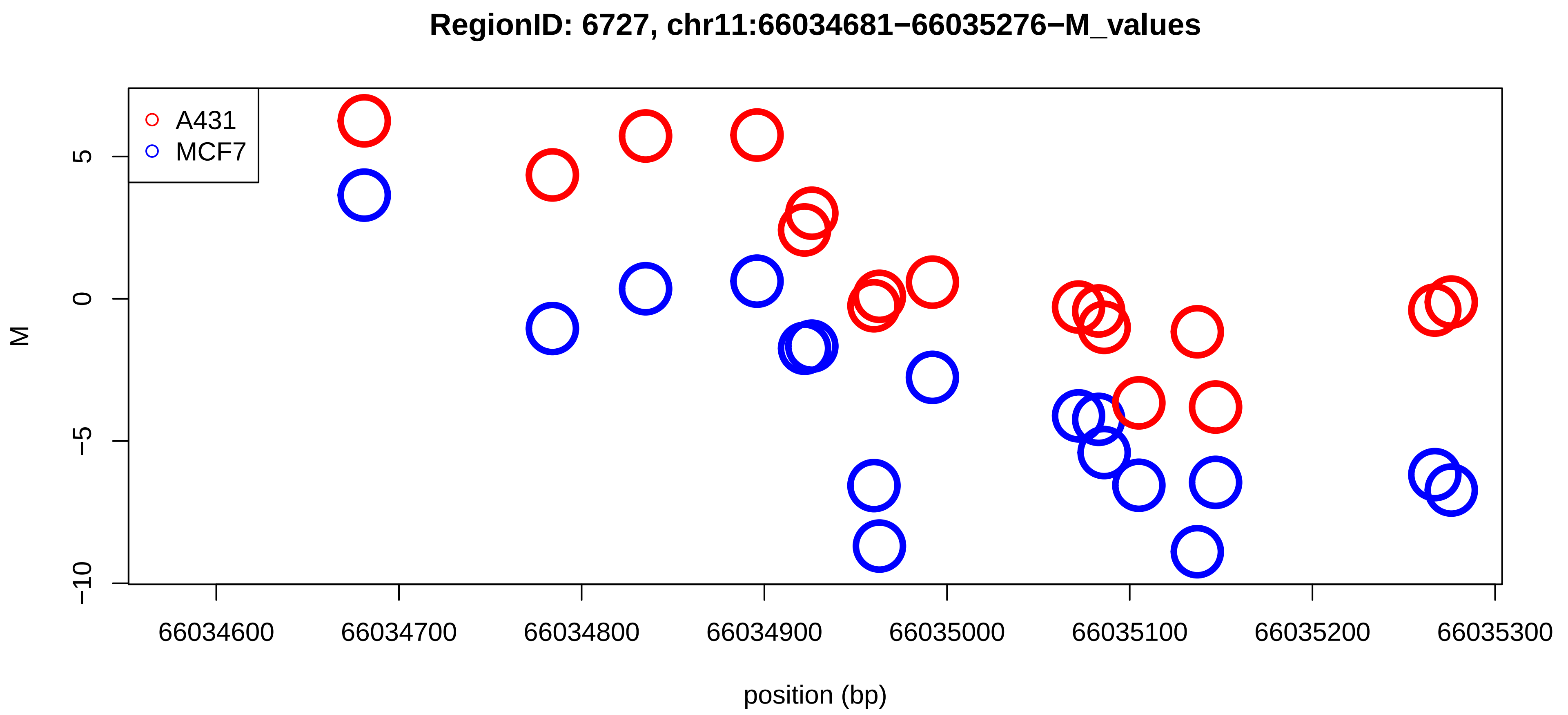

RegionID: 6727, chr11:66034681−66035276−M_values
A431
MCF7
M
5
0
−5
−10
66034600
66034700
66034800
66034900
66035000
66035100
66035200
66035300
position (bp)

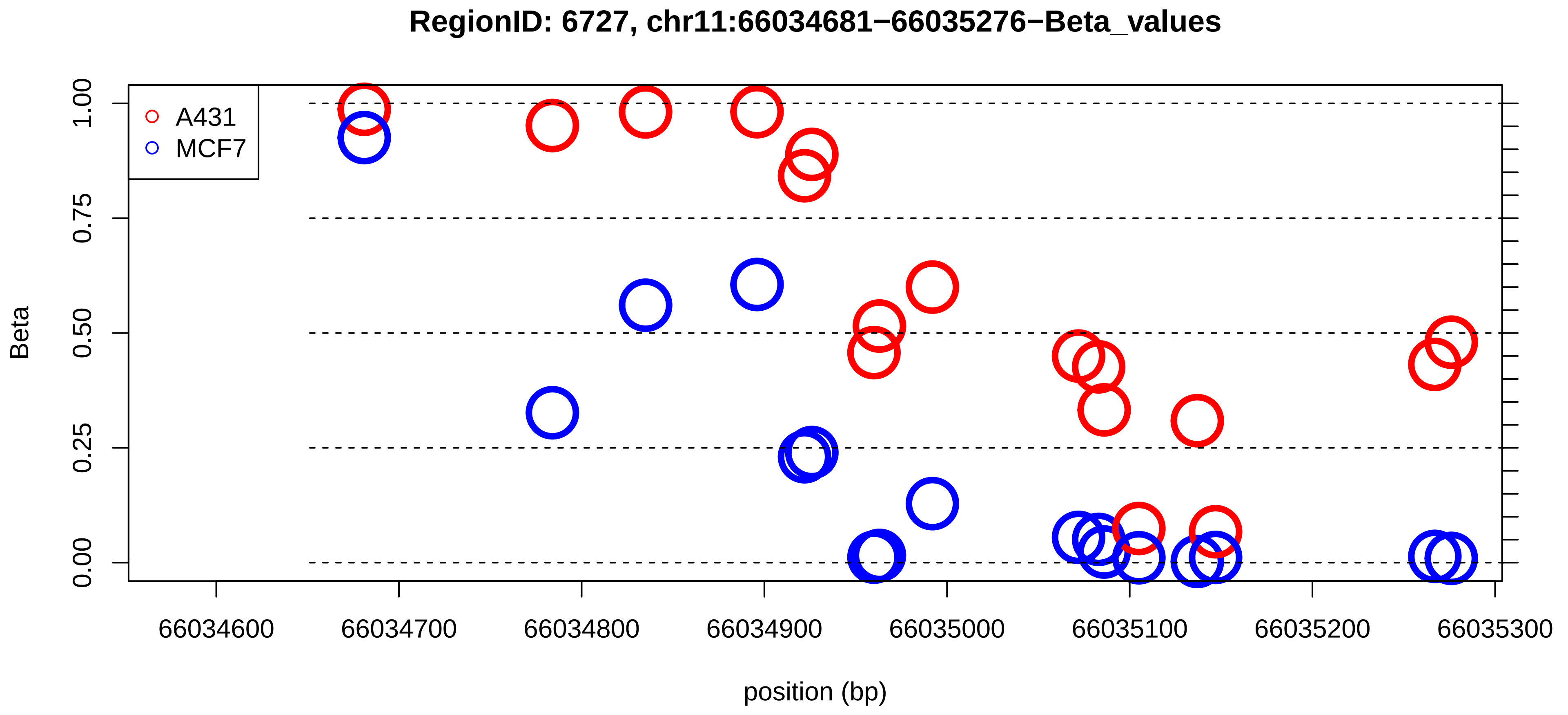

RegionID: 6727, chr11:66034681−66035276−Beta_values
A431
MCF7
Beta
1.00
0.75
0.50
0.25
0.00
66034600
66034700
66034800
66034900
66035000
66035100
66035200
66035300
position (bp)

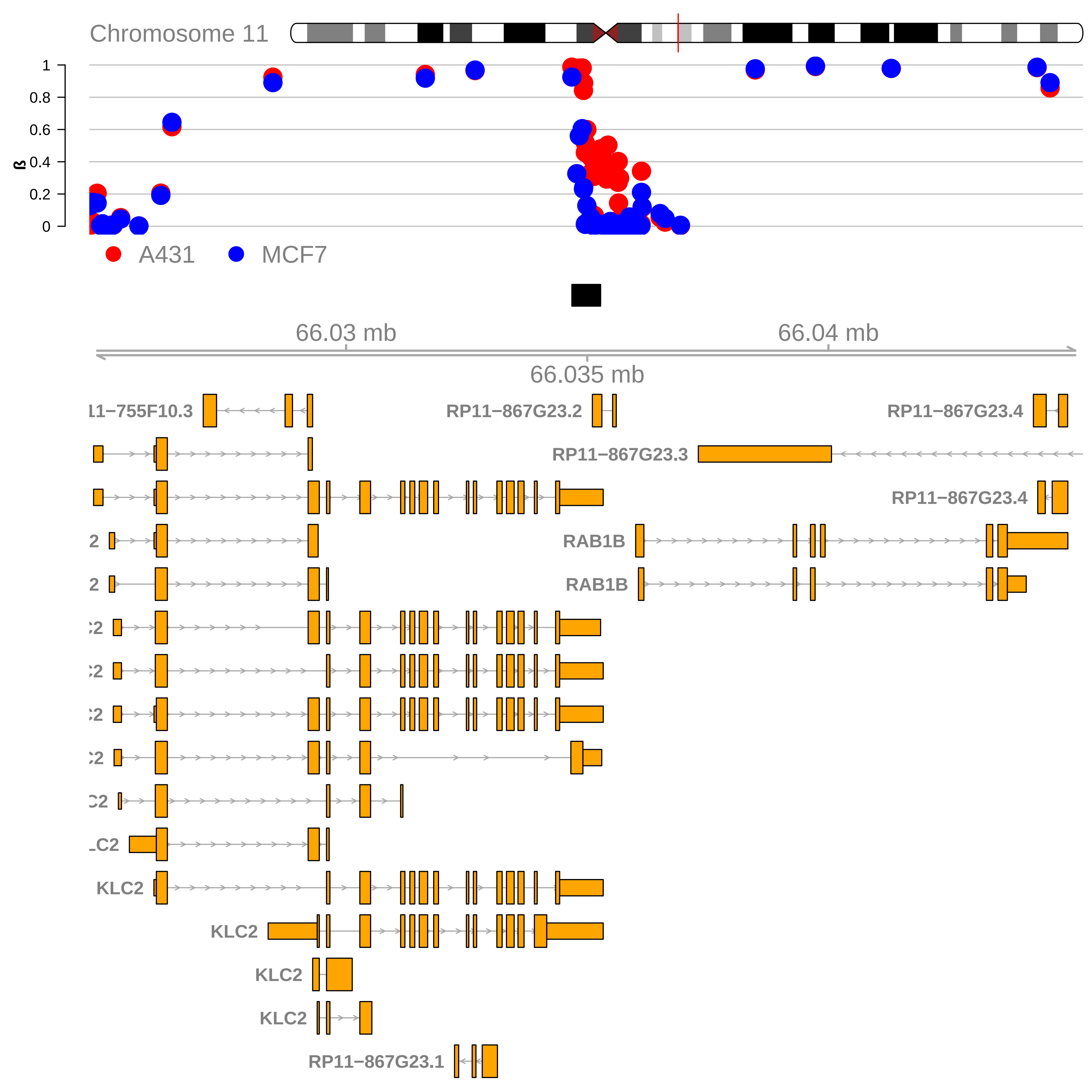

Chromosome 11
1
0.8
0.6
ß 0.4
0.2
0
A431
MCF7
66.03 mb
66.04 mb
66.035 mb
RP11−867G23.2
RP11−867G23.4
RP11−867G23.3
RP11−867G23.4
RAB1B
RAB1B
KLC2
KLC2
KLC2
KLC2
RP11−867G23.1
